# Spatial distribution of the full-length members of the *Grg* family during embryonic neurogenesis reveals a “Grg-mediated repression map” in the mouse telencephalon

**DOI:** 10.1371/journal.pone.0209369

**Published:** 2018-12-20

**Authors:** Charalampos Chrysovalantis Chytoudis-Peroudis, Nikistratos Siskos, Konstantinos Kalyviotis, Ioannis Fysekis, Petros Ypsilantis, Constantinos Simopoulos, George Skavdis, Maria E. Grigoriou

**Affiliations:** 1 Department of Molecular Biology & Genetics, Democritus University of Thrace, Alexandroupolis, Greece; 2 School of Medicine, Democritus University of Thrace, Alexandroupolis, Greece; Oxford Brookes University, UNITED KINGDOM

## Abstract

The full-length members of the Groucho/Transducin-like Enhancer of split gene family, namely *Grg1-4*, encode nuclear corepressors that act either directly, via interaction with transcription factors, or indirectly by modifying histone acetylation or chromatin structure. In this work we describe a detailed expression analysis of *Grg1-4* family members during embryonic neurogenesis in the developing murine telencephalon. *Grg1-4* presented a unique, complex yet overlapping expression pattern; *Grg1* and *Grg3* were mainly detected in the proliferative zones of the telencephalon, *Grg2* mainly in the subpallium and finally, *Grg4* mainly in the subpallial post mitotic neurons. In addition, comparative analysis of the expression of *Grg1-4* revealed that, at these stages, distinct telencephalic progenitor domains or structures are characterized by the presence of different combinations of Grg repressors, thus forming a “Grg-mediated repression map”.

## Introduction

The mammalian telencephalon develops from the alar plate of the secondary prosencephalon and is the most complex region of the brain with respect to both morphology and neuronal phenotypes. Shortly after the closure of the anterior neural tube the interplay between extrinsic signalling molecules induces and sustains the regionalized expression of several transcription factors (TFs), known as patterning factors, that further divide the telencephalon into anatomically and molecularly distinct territories, namely the pallium dorsally and the subpallium ventrally (for review see [1–3]). Patterning factors induce the expression of another set of TFs, known as lineage, or cell fate determinants, which in turn define neuronal identity and cell type specification (for review see [1–3]). For instance Shh emanating initially from the prechordal plate and later from the non evaginated telencephalon, induces in the ventral part of the telencephalon the expression of Gsh1 and Gsh2 (also known as *Gsx1* and *Gsx2*), which then induce the expression of Dlx1, Dlx2 and Ascl1; these three TFs initiate the neuronal differentiation pathways of the subpallial progenitors [4–9]. The same principle of region-specific transcriptional regulation, via the spatially restricted expression of TFs, that underlies the specification of pallial and subpallial identities, is also applied for the subdivision of the pallium and the subpallium into smaller, distinct functional areas. For instance, in the medial part of the subpallium (prospective MGE) Shh induces, along with Gsh1 and Gsh2, Nkx2.1 that promotes the expression of the lineage determinants Lhx6 and Lhx7/8 which are required for the differentiation of globus pallidus (GP) and the generation of the MGE-derived cortical interneurons [10–12]. In addition, Lhx6 is required for the maturation and migration of the MGE derived cortical interneurons by inducing Arx and Cxcr7 expression [11, 12, 13] while Lhx7/8 drives cholinergic neuron fate [11, 14–16]. The importance of strict spatiotemporal regulation of these developmental cascades is best demonstrated in knock-out experiments; for example in murine embryos lacking Nkx2.1, Lhx6 and Lhx7/8 are not expressed, MGE neural progenitors are not specified and the prospective MGE is respecified as LGE, expressing striatal markers such as Pax6 [17–19].

Despite the large number of studies that have focused on the TFs involved in the generation of neuronal progenitors and in the patterning and specification of the telencephalic subdivisions, little is known about the role of transcriptional corepressors in these developmental processes.

The members of the *Gro/TLE/Grg* (*Groucho*/*T**ransducin-**l**ike* *E**nhancer of split/* *G**roucho* *r**elated* *g**ene*) gene family encode nuclear proteins that act as transcriptional corepressors and regulate several signalling pathways, including the Notch, the Wnt and the BMP/TGF-β pathways [20–27]. The prototype of the family, namely Groucho (Gro), was characterized in *Drosophila* as a corepressor initially of the Hairy-related proteins, and later of Engrailed, Dorsal and Runt domain proteins [20–28].

In mammals the *Gro/TLE/Grg* family consists of four members (*TLE1-4* for human, *Grg1-4* for mouse) which code for transcriptional corepressors of similar size and structure to the *Drosophila* Gro protein [20–22, 24–28]. Grg1-4/TLE1-4 proteins consist of five domains; the amino terminal Q (glutamine rich) and the carboxyterminal WD (tryptophane—aspartic acid tandem repeats) domains are highly conserved, while the three internal domains, namely the SP (serine-proline rich), the GP (glycine-proline rich) and the CcN (putative cdc2 kinase and protein kinase CK2 phosphorylation sites and the nuclear localization sequence), are less conserved [20–27]. The Q, the WD, the SP and the GP domains mediate protein to protein interactions between the Grg/TLE corepressors and several transcription factors and cofactors [21–23, 25, 27, 29]. Moreover, the Q domain conveys the oligomerization of the TLE1-4/Grg1-4 proteins required for repression of several Grg1-4/TLE1-4 target genes [21–23, 25–27, 30] while the GP domain interacts with histone deacetylase [21, 22, 25, 27, 31]. Finally, the CcN domain contains the nuclear localization sequence as well as the cdc2 and the protein kinase CK2 phosphorylation sites [21, 23, 25, 27].

Grg1-4/TLE1-4 proteins mediate repression either directly, interacting with transcription factors, or indirectly modifying histone acetylation or chromatin structure [21, 32, 33, 34, 23, 25, 27]. For example the interaction between Grg4 and Pax2 inhibits Pax2 phosphorylation by JNK suppressing Pax mediated transcriptional activation [35]. A different mechanism has also been described for Grg4 repression of Pax2-dependent activation [36]. More specifically, the interaction of Grg4 with Pax2 results in the displacement of the adaptor protein PTIP, component of the KMT2C/D (Mll3/4) histone H3 lysine 4 (H3K4) methylation complex, inhibiting H3K4 methylation required for Pax mediated transcriptional activation [36, 37]. Moreover, Grg3 interacts with the DNA binding protein FoxA1 and binds nucleosomal arrays; this interaction promotes condensation into higher order chromatin leading to region-specific gene silencing [38].

In mammals two shorter proteins homologous either to the amino terminal (Aminoterminal Enhancer of split, AES or Grg5/TLE5) or to the carboxyterminal (Grg6/TLE6) region of the Gro protein have also been characterized [20, 21, 25, 26, 29, 39]. Grg5/AES/TLE5 and Grg6/TLE6 are truncated family members, consisting of the Q and GP domains or the WD domain respectively [29, 21, 22, 25, 27, 30] and are expressed from two different loci [25, 29, 40,41]. Grg5/AES/TLE5 and Grg6/TLE6 are thought to act as dominant negative forms of the Grg 1-4/TLE1-4 corepressors. For instance it has been shown *in vitro* that Grg5 reduces Grg4-mediated Nkx repression [42].

The expression of mouse *Grg1-4* has been analyzed in a number of studies performed when these genes were first characterized and the overall pattern of their expression in the embryo was described [43–47]. Comparative analysis of *Grg1-4*, however, has only been performed in the developing spinal cord and pancreas; these experiments showed that *Grg1-4* are expressed with unique, overlapping patterns and mediate the activity of the TFs implicated in the development of these organs [42,48]. Several transcription factors that directly interact with Grg1-4/TLE1-4 have been identified so far; interestingly each family member is characterized by distinct interaction repertoire [20–22, 24–27, 29, 32, 42,48–50]. Among the TFs that have a crucial role in telencephalic development, several have been shown to interact, in other systems, with Grg 1-4/TLE1-4 (for example Arx and Foxg1; [48–51]); others are homologous to TFs that interact with members of the Grg /TLE family (for example Nkx2.1 [42, 43]). Finally, in the developing telencephalon of *Xenopus*, Grg2 has been implicated in the proper patterning of the ventral telencephalon [51]. These data prompted us to study the spatial distribution of *Grg 1–4* in the murine telencephalon during embryonic neurogenesis, as no comprehensive description of their patterns of expression has been described so far. We report a detailed, comparative spatiotemporal analysis of the expression of *Grg1-4* during embryonic neurogenesis (between E11.5—E15.5) in the developing telencephalon. Our data show that *Grg1-4* are expressed with a unique yet overlapping expression pattern; distinct progenitor domains and structures within the murine telencephalon are characterized by the expression of different combinations of Grg repressors forming a “Grg mediated repressor map”.

## Materials and methods

### Animals

All experiments were conducted in accordance with the European Communities Council Directives of 24 November 1986 (86/609/EEC) and of September 22, 2010 (10/63/EU), which was the legislation in force at the time of experimentation. The protocols were approved by the committee for the Care and Use of Laboratory animals of our Institution and of the Prefecture of Evros, Thrace, Greece (permits T/1571/13.5.09 and 5042/3-4-2013).

### Tissue preparation, fixation and sectioning

Embryos: C57BL/6J wild-type mice were bred in the animal facility of the Laboratory of Experimental Surgery and Surgical Research of the School of Medicine of Democritus University of Thrace. Time mated pregnant female mice (C57/BL6) were euthanized with carbon dioxide at specific time-points (E11.5, E12.5, E13.5 and E15.5; the day of vaginal plug detection was considered as day 0.5) and embryos were dissected free of maternal tissues in cold phosphate-buffered saline (PBS, pH 7.4).

For non radioactive *in situ* hybridization embryos were fixed in 4% w/v paraformaldehyde (PFA) for 24 h at 4 ^o^C or in Z7 for 1–3 [52,53], washed with PBS, cryoprotected in 30% w/v sucrose in PBS, embedded in Tissue Freezing Medium (Leica Microsystems) and sectioned at 12 μm using a cryostat (Leica 1900UV). Sections were transferred to superfrost plus slides (Fisher Scientific), air dried for at least 30 min and stored at -80 ^o^C until later use.

For cultures of primary embryonic cells, the subpallium of the embryonic brain were carefully dissected and incubated in DMEM/F12 (GIBCO) supplemented with B27 (GIBCO), 2mM GlutaMax (GIBCO), 15mM Hepes (GIBCO) and 0.05mg/ml Gentamicin (GIBCO) at 37°C for 15 min. Upon mechanical trituration, cells were plated on cultured O/N on glass coverslips coated with poly-D-lysine, placed in the bottom of the plate (Sigma, Germany).

#### *In situ* hybridization on mouse embryo tissue sections

*In situ* hybridization on sections was performed as previously described [52,53].

RNA probes were synthesized by *in vitro* transcription with T3 or T7 RNA polymerase (Takara), according to manufacturer’s instructions, using Digoxigenin-11-UTP (Roche). For *Grg1* a 609 bp fragment (nt 16–615 of the mouse cDNA, NM_001285529) was used. For *Grg2* a 890 bp fragment (nt 791–1681 of the mouse cDNA- NM_019725) was used. For *Grg3* a 610 bp fragment (nt 1166–17775 of the mouse cDNA NM_001083927) was used. For *Grg4* a 1042 bp fragment (nt 2756-3797of the mouse cDNA- NM_011600) was used. Probes were designed to hybridize with abundant mRNA variants of each gene. For *Nkx2*.*1* a 2832bp fragment (nt 597–2832 of the mouse cDNA- NM_009385) was used. For *Lhx6*, a 1341bp fragment (nt 1464–2805 of the mouse cDNA- NM_008500) was used. For *Lhx7/8*, an 163 bp fragment (nt 1022–1185 of the mouse cDNA- NM_010713.2) was used [52,53]. For *Arx* a 873 bp fragment (nt 1969–2842 of the mouse cDNA- NM_007492) was used. For *Gsh2* a 704 bp fragment (nt 464–1168 of the mouse cDNA- NM_133256.2) was used. For *Dlx2* a 1731 bp fragment (nt 760–2490 of the mouse cDNA—NM_010054.2) was used.

For the expression analysis of *Grg1-4* as well as for the comparative analysis, three independent embryos of different litters were used for each *in situ* hybridization experiment at each embryonic age studied. All experiments were repeated twice.

### Immunofluorescence on primary embryonic cells of the MGE

For immunofluorescence on cells the coverslips were washed in PBT (0.1% v/v Triton X-100 in PBS), blocked in 1% v/v FCS and 0.1% w/v BSA in PBT for at least 1 h at room temperature (RT), and subsequently incubated with primary antibodies, diluted in blocking solution, for 14–16 h at 4°C. Slides were washed in PBS (3 washes, 10 min each) and incubated with the secondary antibody, diluted in blocking solution, for 2 h at room temperature. Slides were washed in PBS (3 washes, 5 min each) and mounted in Everbright mounting medium (Biotium, USA). The following antibodies were used: anti-Tle4 (1: 250, Santa Cruz), anti-Tle2 (1: 250, Santa Cruz), anti Nkx2.1 (1: 250, Santa Cruz), anti-Lhx6 and anti-Lhx7/8 prepared by our lab [53], anti-rabbit IgG CF488A (1:500, Biotium) and anti-mouse IgG CF568 (1:500, Biotium). Three independent experiments with duplicate samples were performed. In each experiment, following immunofluorescence, 1000 cells were scored. Values represent the mean ± SEM of the immunoreactive cell number, expressed as the percentage of total cell counts from 3 independent experiments with duplicate samples. Statistical analyses were performed using Student's t-test. *P<0.05.

### Microscopy

The sections or the coverslips were analysed using a D-6000 upright microscope (Leica, Germany). Images were captured with the camera software (Leica) and assembled in Adobe Photoshop.

## Results

To determine the expression pattern of *Grg1-4* in the telencephalon we performed *in situ* hybridization experiments on sections of embryonic head between E11.5 and E15.5, a period that coincides with the peak of embryonic neurogenesis in the mouse. To rule out cross-hybridization each probe was carefully selected following multiple alignment analysis of all the mRNA sequences of the *Grg* family members. Moreover, control experiments using *Grg1-4* sense probes did not show any specific hybridization confirming the specificity of the method (S1 Fig).

### *Grg1* is expressed at various levels mainly in the ventricular zone of the telencephalon

**Fig 1:**
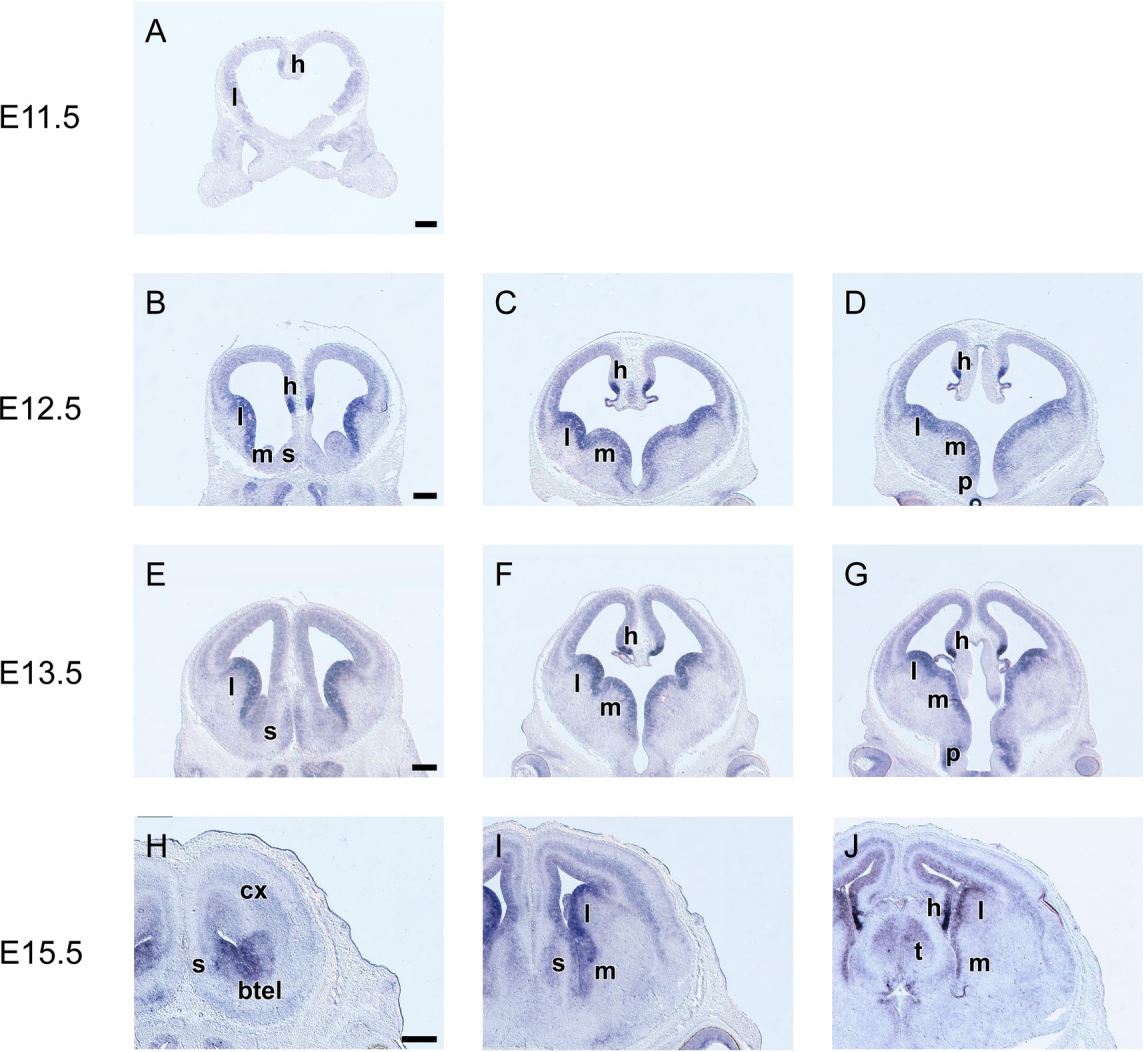
*In situ* hybridization on coronal sections through the embryonic telencephalon from rostral (left) to caudal (right) levels hybridized with an antisense RNA probe of *Grg1* at E11.5 (A), E12.5 (B—D), E13.5 (E‐I) and E15.5 (H—J) m: MGE, l: LGE, l: LGE, s: septum, btel: basal telencephalon, cx: cortex. Scale: 250 μm.

**Fig 2:**
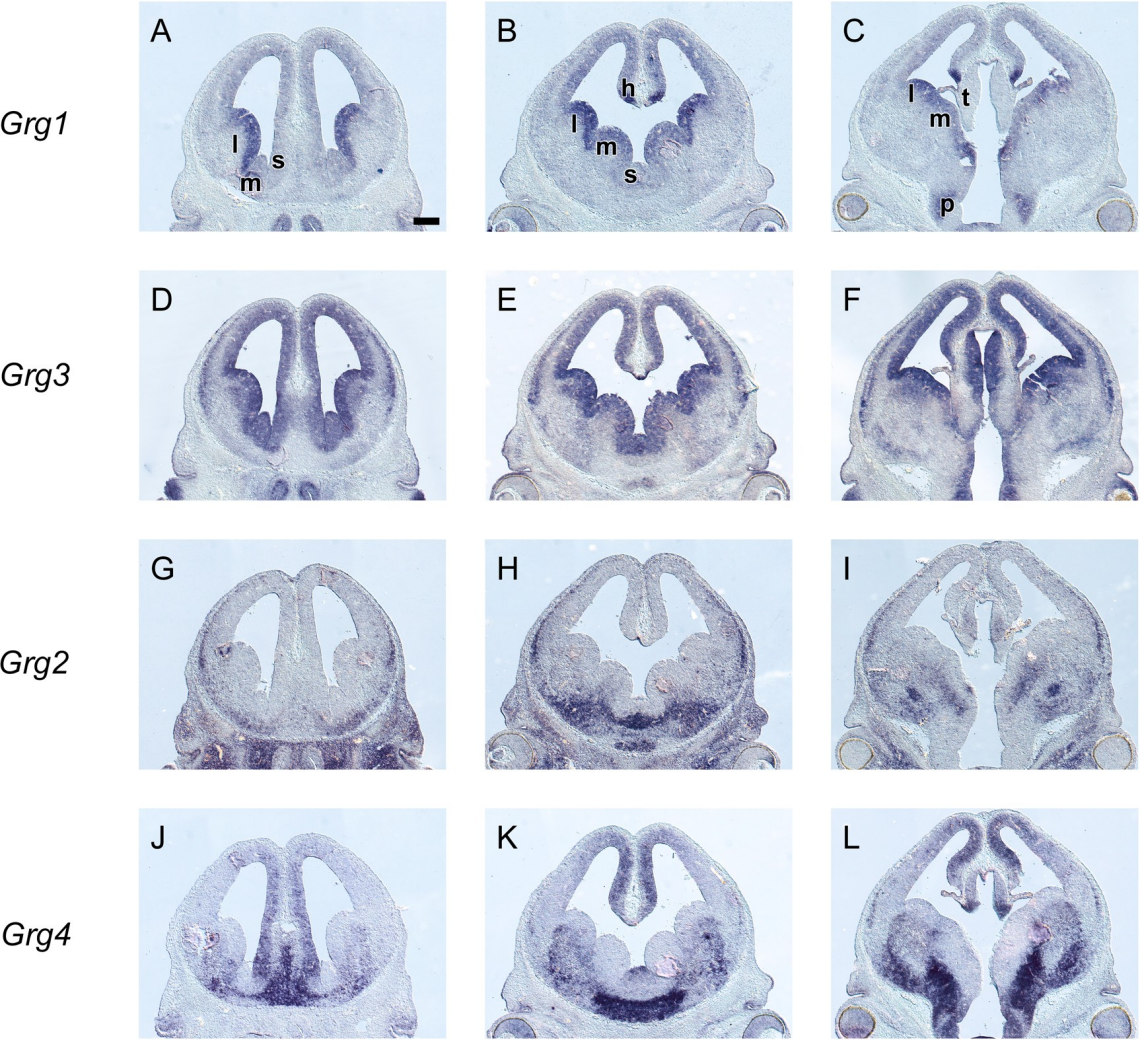
*In situ* hybridization on adjacent coronal sections through the embryonic telencephalon at E13.5 from rostral (left) to caudal (right) levels hybridized with an antisense RNA probe of *Grg1* (A—C), *Grg3* (D—F), *Grg2* (G‐I) and *Grg4* (J—L) m: MGE, l: LGE, l: LGE, s: septum. Scale: 250μm.

Low levels of *Grg1* expression were first detected at E11.5 in the ventricular zone (VZ, zone of proliferation) of the prospective lateral ganglionic eminence (LGE) and of the cortical hem (Fig 1A). At E12.5 *Grg1* expression in the VZ of the cortical hem and of the LGE persisted and was upregulated (Fig 1B–1D). Moreover, the expression domain of *Grg1* expanded ventrally into the VZ of the medial ganglionic eminence (MGE) and dorsally in the VZ of the pallium (Fig 1B–1D). In the pallium *Grg1* mRNA levels were low and uniformly distributed (Fig 1B–1D). Within the MGE the expression of *Grg1* mRNA formed a gradient (Fig 1B–1D) with low expression levels in the rostroventral part of the MGE. This pattern of expression was also observed at E13.5 (Fig 1E–1G, Fig 2A–2C); in addition at this stage *Grg1* mRNA was also detected in the subventricular zone (SVZ, the second zone of proliferation) of the preoptic area (PO) and in few cells in the SVZ of the LGE (Fig 1E–1G). This pattern of expression persisted at E14.5. At E15.5 low levels of *Grg1* expression were detected in the VZ of the MGE and of the pallium as well as in the SVZ of the preoptic area (Fig 1H–1J); higher expression levels were observed in the VZ of the LGE (Fig 1I), the striatal septum (Fig 1H) and of the cortical hem (Fig 1J). Finally, at these stages *Grg1* mRNA was also detected in post mitotic cell populations of the developing cortex, namely in the outer cortical plate (CP), in the subplate (SP), and in the intermediate zone (IZ, Fig 1H–1J).

### *Grg2* is mainly expressed in the ventral subpallium and its derivatives

**Fig 3:**
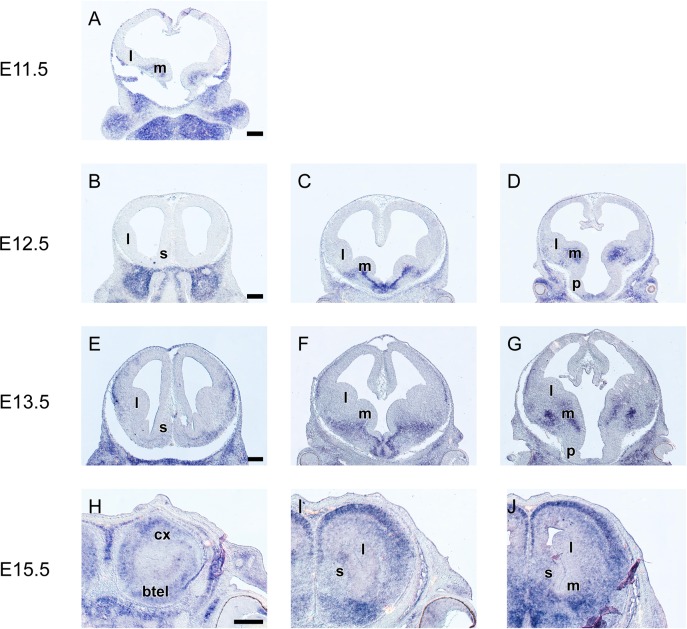
*In situ* hybridization on coronal sections through the embryonic telencephalon from rostral (rostral (left) to caudal (right) levels hybridized with an antisense RNA probe *Grg2* at E11.5 (A), E12.5 (B—D), E13.5 (E‐I) and E15.5 (H—J) m: MGE, l: LGE, l: LGE, s: septum. Scale: 250 μm.

*Grg2* expression first appeared at late E11.5 in the SVZ of the MGE, shortly after the emergence of the eminences from the basal telencephalic wall (Fig 3A). One day later, at E12.5 *Grg2* mRNA was observed in the SVZ and in the mantle zones of the MGE (Fig 3C and 3D). Moreover, at this stage *Grg2* was also detected in the VZ, SVZ and the mantle of the preoptic area and of the pallidal septum (Fig 3B–3D). At E13.5 *Grg2* mRNA was detected in the SVZ and in the mantle zone of the MGE; however at this stage the expression in the SVZ formed a gradient with barely detectable levels at the rostroventral part of the MGE (Fig 3E–3G). Moreover at this stage, *Grg2* mRNA was detected in the mantle zone of the MGE (anlage of the globus pallidus, ventral pallidum, caudoventral domain), in the mantle zone of the LGE, in the SVZ and mantle of the preoptic area and of the pallidal septum, as well as in the ventralmost part of the cortical plate (Fig 2G–2I, Fig 3E–3G). This pattern persisted at E14.5. At E15.5 *Grg2* expression was detected only in post mitotic populations in the septum, in the ventral pallidum and in the globus pallidus (Fig 3H–3J). In addition, strong expression was observed in the CP, from the insular to the hippocampal anlage; weak expression was also detected in cells of the relevant SP and of the IZ (Fig 3H–3J).

### *Grg3* is broadly expressed in the proliferative zones of the telencephalon

**Fig 4:**
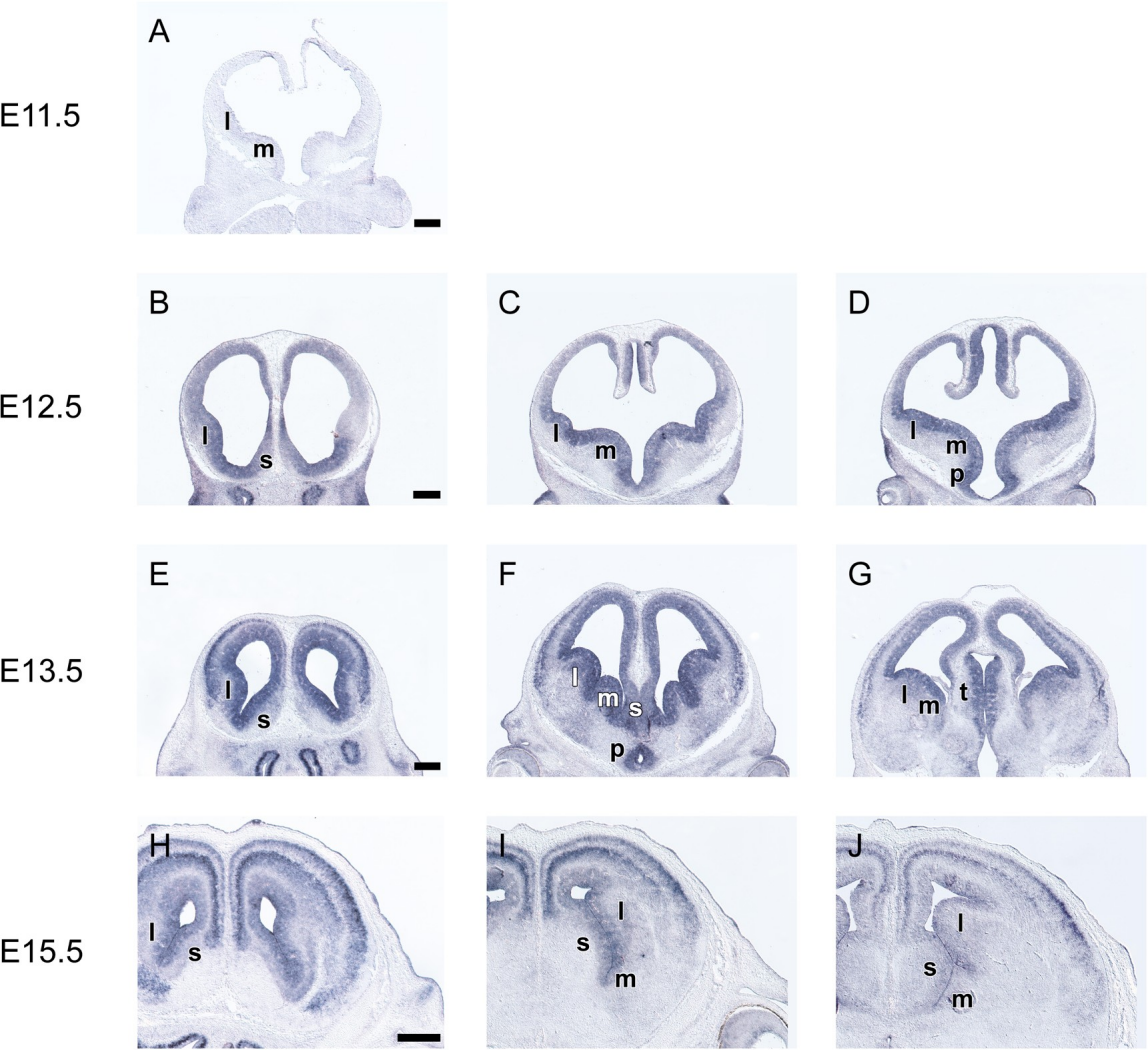
*In situ* hybridization on coronal sections through the embryonic telencephalon from rostral (left) to caudal (right) levels hybridized with an antisense RNA probe *Grg3* at E11.5 (A), E12.5 (B—D), E13.5 (E‐I) and E15.5 (H—J) m: MGE, l: LGE, l: LGE, s: septum. Scale: 250 μm.

Very low levels of *Grg3* expression were first detected at E11.5 in the VZ of the subpallium and of the neighbouring pallium (Fig 4A). One day later, at E12.5 *Grg3* expression was detected throughout the VZ of the pallium and of the subpallium (Fig 4B–4D). In the subpallial VZ *Grg3* was highly and uniformly expressed; in the pallium though, it formed a ventral to dorsal gradient (Fig 4B–4D). In subsequent stages, at E13.5 and E14.5, *Grg3* mRNA was highly and uniformly expressed in the VZ of the pallium and of the subpallium, as well as in the SVZ of the LGE. In addition *Grg3* was expressed in post mitotic cells of the telencephalon, in the CP and, at low levels, in the mantle zone of the MGE (Fig 2D–2F, Fig 4E–4G). At E15.5 *Grg3* mRNA was uniformly expressed in the VZ of the telencephalon, as well as in the outer CP and in the SP (Fig 4H–4J).

### *Grg4* is expressed mainly in post mitotic cells of the subpallium

**Fig 5:**
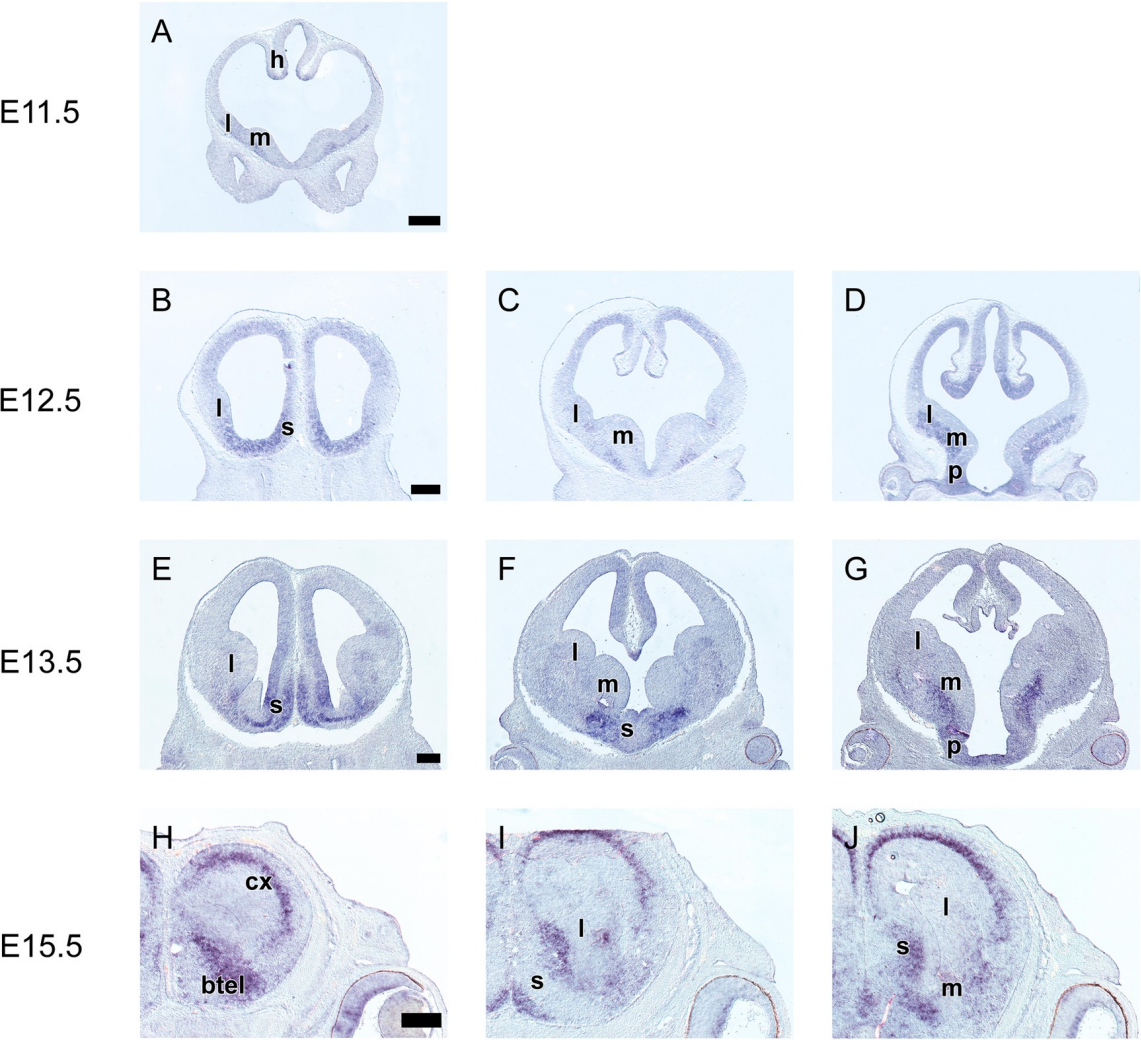
*In situ* hybridization on coronal sections through the embryonic telencephalon from rostral (left) to caudal (right) levels hybridized with an antisense RNA probe *Grg4* at E11.5 (A), E12.5 (B—D), E13.5 (E‐I) and E15.5 (H—J) m: MGE, l: LGE, l: LGE, s: septum. Scale: 250 μm.

Weak *Grg4* expression was first detected in the telencephalon at E11.5 in the newly formed mantle layer of the ganglionic eminences (Fig 5A). At E12.5 *Grg4* mRNA was detected in the VZ of the dorsal septum and of the PO as well as in the mantle zone of the LGE and the MGE (Fig 5B–5D). One day later, at E13.5 this pattern persisted, however at this stage *Grg4* mRNA was also detected in the SVZ of the LGE, in the SVZ and mantle zones of the ventral septum and in the mantle zone of the PO (Fig 2J–2L,Fig 5E–5G). At E15.5 *Grg4* mRNA was detected in the mantle layer of the septum in the basal telencephalon as well as in the CP of the pallium (Fig 5H–5J). Notably within the septum two domains of Grg4 expression were detected separated by a *Grg4* negative corridor (Fig 5H–5J).

### Comparative analysis reveals a complex pattern with distinct cell populations characterized by the combined expression of members of the *Grg* family

Our analysis of the spatiotemporal expression pattern of the full-length *Grg* family members showed that these genes are expressed with a characteristic pattern; in fact our results so far suggested that in several brain regions more than one members of the *Grg* family may be expressed, thus we then performed *in situ* hybridization experiments on sagittal and coronal serial sections at E13.5, a stage important for the patterning and regionalization of the mammalian telencephalon.

**Fig 6:**
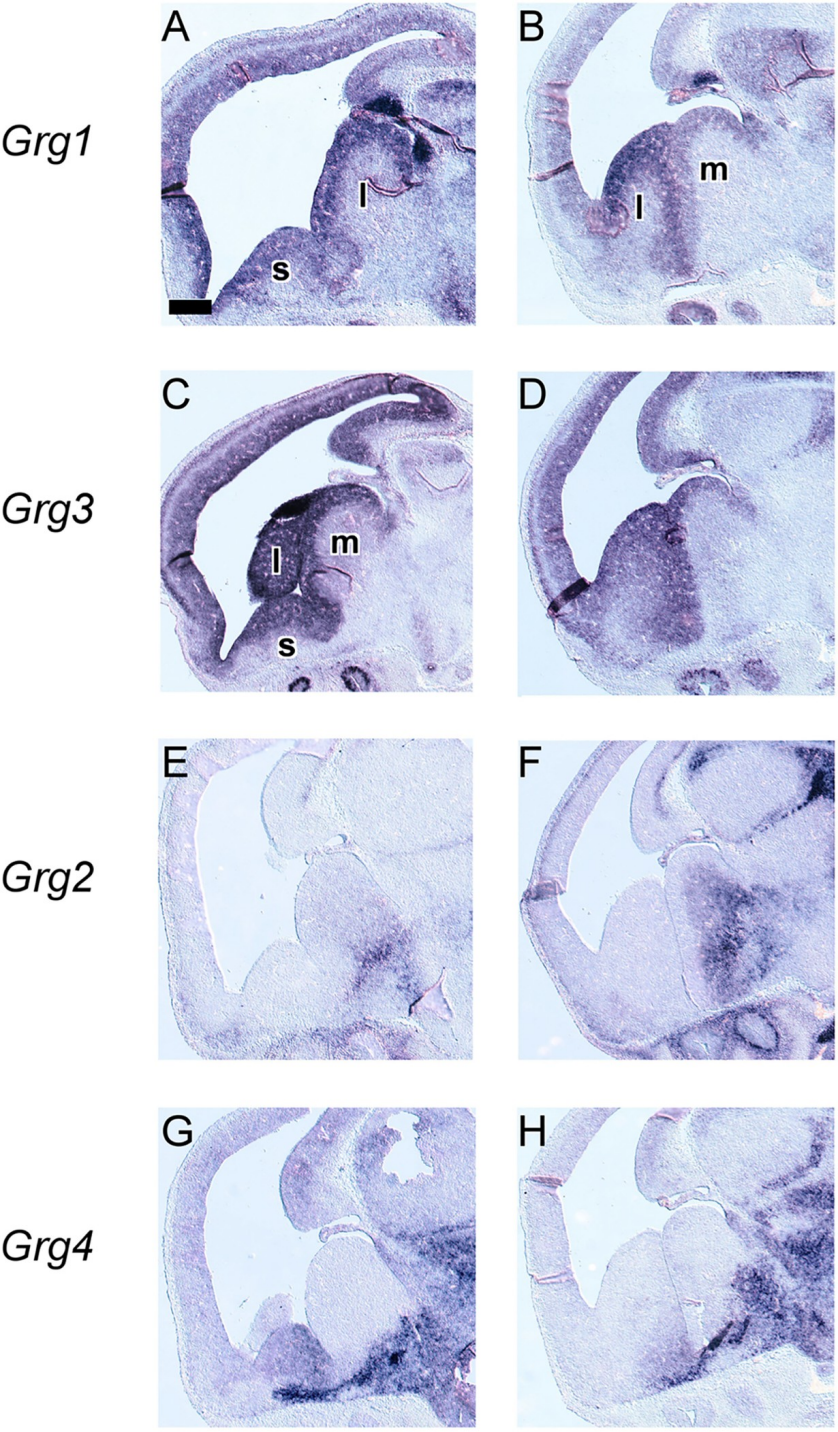
*In situ* hybridization on adjacent sagittal sections through the embryonic telencephalon from lateral (left) to medial (right) levels hybridized with an antisense RNA probe of Grg1 (A, B), Grg3 (C, D), Grg2 (E,F) and Grg4 (G, H) m: MGE, l: LGE, l: LGE, s: septum. Scale: 200 μm.

Within the pallium *Grg1* and *Grg3* were coexpressed in the VZ of the cortical hem and of the lateral and the dorsal pallium (Fig 2B–2E, Fig 6A–6D). The VZ of the medial and the dorsal-most pallium was characterized by the expression of *Grg3* and *Grg4* and low levels of *Grg1* (Fig 2D–2F and Fig 2J–2L). In addition overlapping domains of expression were observed for *Grg2*, *Grg3* and *Grg4* in the MZ and in the outer CP of the lateral and of the dorsal pallium (Fig 2F, 2I and 2L).

**Fig 7:**
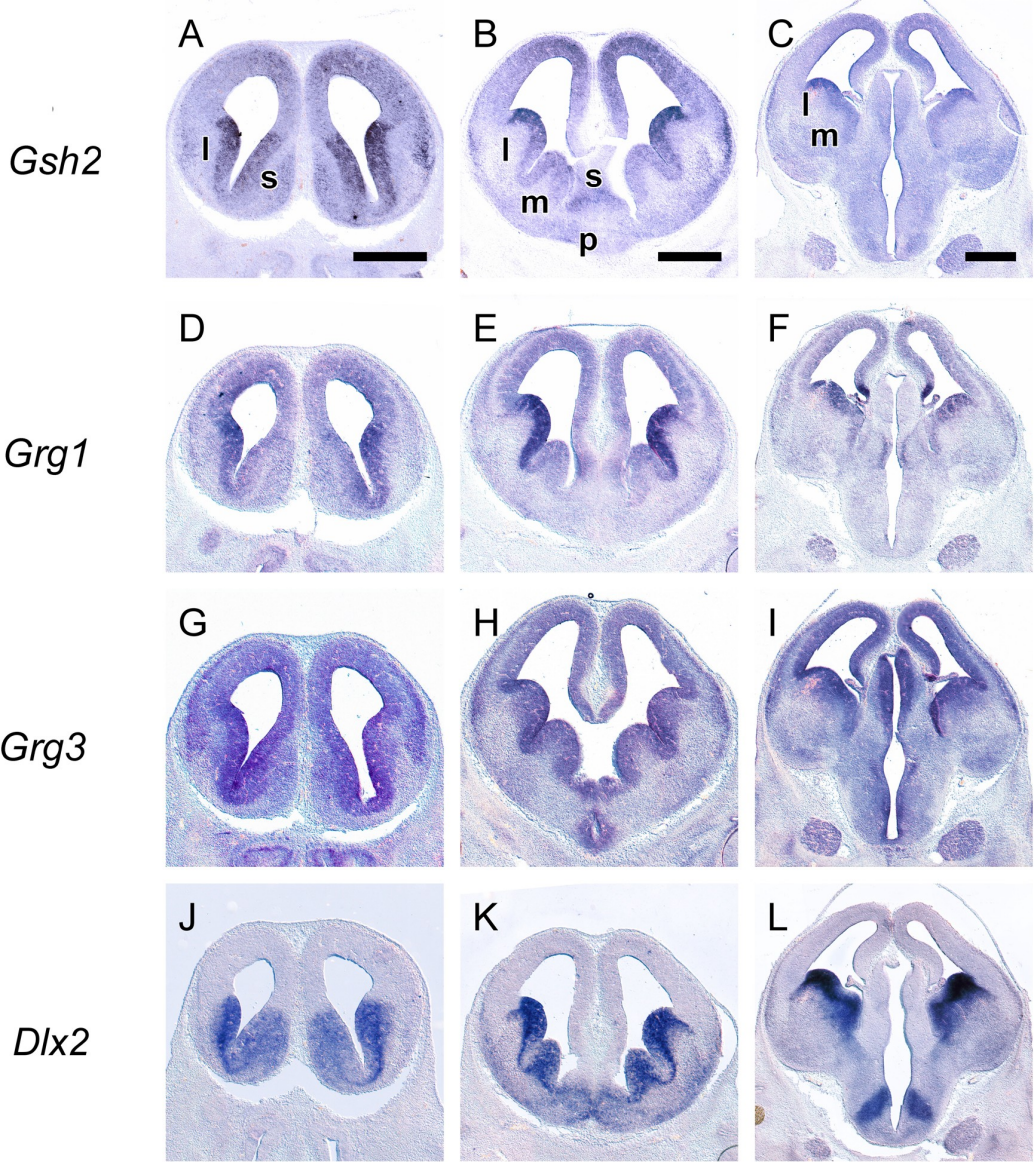
*In situ* hybridization on adjacent coronal sections through the embryonic telencephalon from rostral (left) to caudal (right) levels hybridized with an antisense RNA probe of Gsh2 (A—C), Grg1 (D—F), Grg3 (G‐I) and Dlx2 (J—L) m: MGE, l: LGE, l: LGE, s: septum, p: preoptic area. Scale: 500 μm.

In the ganglionic eminences, a more complex pattern was detected so we used, along with *Grg1-4*, a number of well characterized markers, namely, *Gsh2*, *Dlx2*, *Nkx2*.*1*, *Arx*, *Lhx6* and *Lhx7/8*. The VZ of the ganglionic eminences was characterized by the combinatorial expression of *Grg1* and *Grg3*; high levels of both mRNAs were detected in the VZ of the LGE (Fig 2A–2C, 2D–2F, Fig 6A–6D, Fig 7D–7I). In the VZ of the MGE, *Grg3* and *Grg1* were also expressed (Fig 2A–2C to Fig 2D–2F). Interestingly the the mRNA of *Grg1* was not uniformly distributed–it was barely detected in the rostroventral part but gradually its levels increased in the posterior and dorsal aspects (Fig 2A–2C to Fig 2D–2F, Fig 6A–6D); this pattern of expression was similar to that of *Gsh2* (compare Fig 2A–2C to Fig 2D–2F, Fig 6A–6D and Fig 7A–7C to Fig 7D–7F, Fig 7G–7I and Fig 7J–7L).

**Fig 8:**
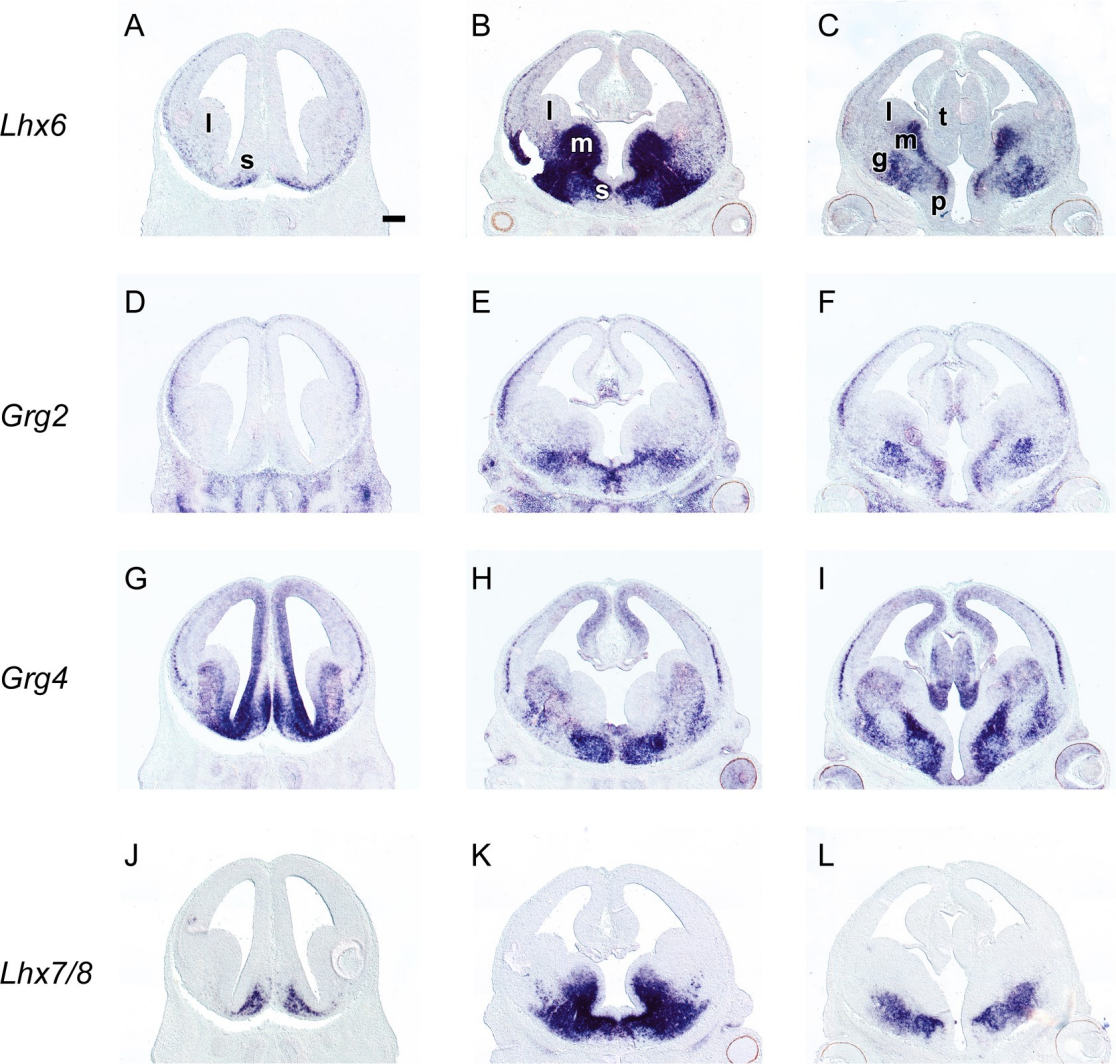
*In situ* hybridization on adjacent coronal sections through the embryonic telencephalon at E13.5 from rostral (left) to caudal (right) levels hybridized with an antisense RNA probe of *Lhx6* (A—C), *Grg2* (D—F), *Grg4* (G‐I) and *Lhx7/8* (J—L) m: MGE, l: LGE, l: LGE, s: septum. Scale: 250 μm.

**Fig 9:**
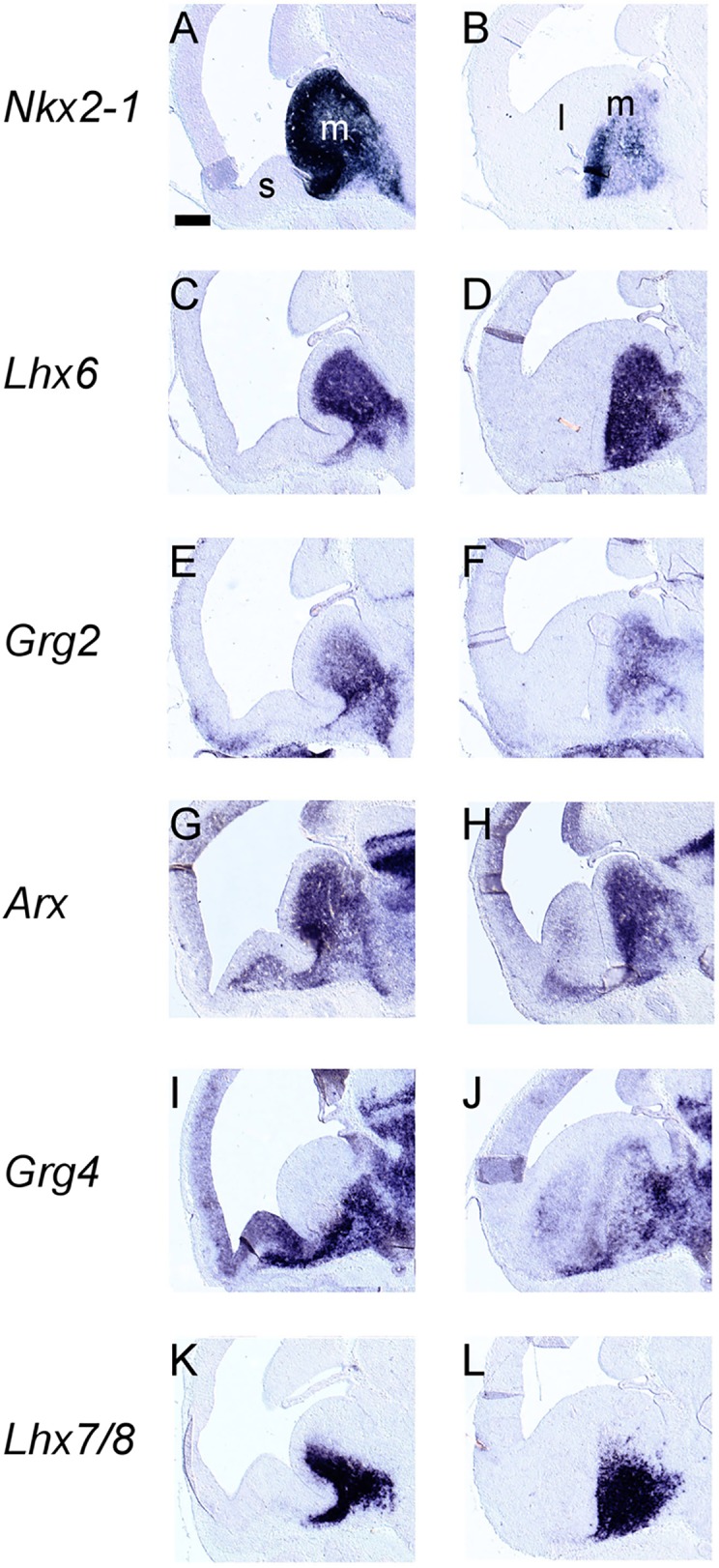
*In situ* hybridization on adjacent sagittal sections through the embryonic telencephalon from lateral (left) to medial (right) levels hybridized with an antisense RNA probe of *Nkx2*.*1* (A, B), *Lhx6* (C, D), *Grg2* (E,F) and *Arx* (G, H), *Grg4* (I,J) and *Lhx7/8* (K,L) m: MGE, l: LGE, l: LGE, s: septum. Scale: 250 μm.

In the SVZ, different territories expressed different combinations of *Grg* family members; *Grg4* and low levels of *Grg3* were detected in the SVZ of the LGE (Fig 2D–2F and 2J–2L, Fig 6C, 6D, 6G and 6H, Fig 7H, Fig 8G–8I and Fig 9J). *Grg2* was detected in the SVZ of the MGE with low levels in the rostroventral fields (Fig 2E and 2F and Fig 6E and 6F) clearly defined upon comparison with the expression patterns of *Lhx6* and *Lhx7/8* within this zone (compare Fig 8B, 8C, 8K, 8L with 8E and 8F). Interestingly, partially overlapping expression of *Grg2* and *Grg4* was observed in the SVZ of the caudoventral MGE (Fig 8H, 8I, 8K and 8L, Fig 6E–6H), in a subpopulation that also expressed *Nkx2*.*1*, *Lhx6*, *Arx and Lhx7/8* (Fig 8C, 8F, 8I and 8L and Fig 9 compare 9A–9D, 9G, 9H, 9K, 9L with 9E, 9F, 9I and 9J). The mantle zone of the LGE was characterized by the expression of *Grg4*; notably the mantle zone of the MGE was characterized mainly by the expression of *Grg2* (Fig 2G–2L, Fig 6E–6H), however cell populations expressing *Grg4* were also detected (Fig 9E and 9I). The comparison of the expression pattern of *Grg2* and *Grg4* within the MGE with the patterns of *Arx*, *Lhx6* and *Lhx7/8* revealed that in the globus pallidus anlage only *Grg2* mRNA was detected (Fig 8 compare 8F to 8I) while the ventral pallidum, the caudate-putamen and the caudoventral MGE expressed both *Grg2* and *Grg4* (Fig 8D–8I and Fig 9).

In the PO, *Grg3* and *Grg4* were expressed in the VZ (Fig 2F and 2L). In the SVZ and mantle zone of the PO overlapping cell populations expressing *Grg1*, *Grg2* and *Grg4* mRNAs were observed (Fig 2C and 2L, Fig 8F and 8I). Finally, in the septum, that consists of both pallial and subpallial territories *Grg3* and *Grg4* mRNA were detected in the VZ with high levels mainly at the striatal fields (Fig 2D and 2J and Fig 6C and 6G). In the SVZ and the mantle of the ventralmost part of the septum *Grg2* and *Grg4* were coexpressed at rostral striatal fields (Fig 8D and 8G), yet at caudal pallidal levels their patterns appeared complementary (Fig 8E and 8H and Fig 9E and 9I).

**Fig 10:**
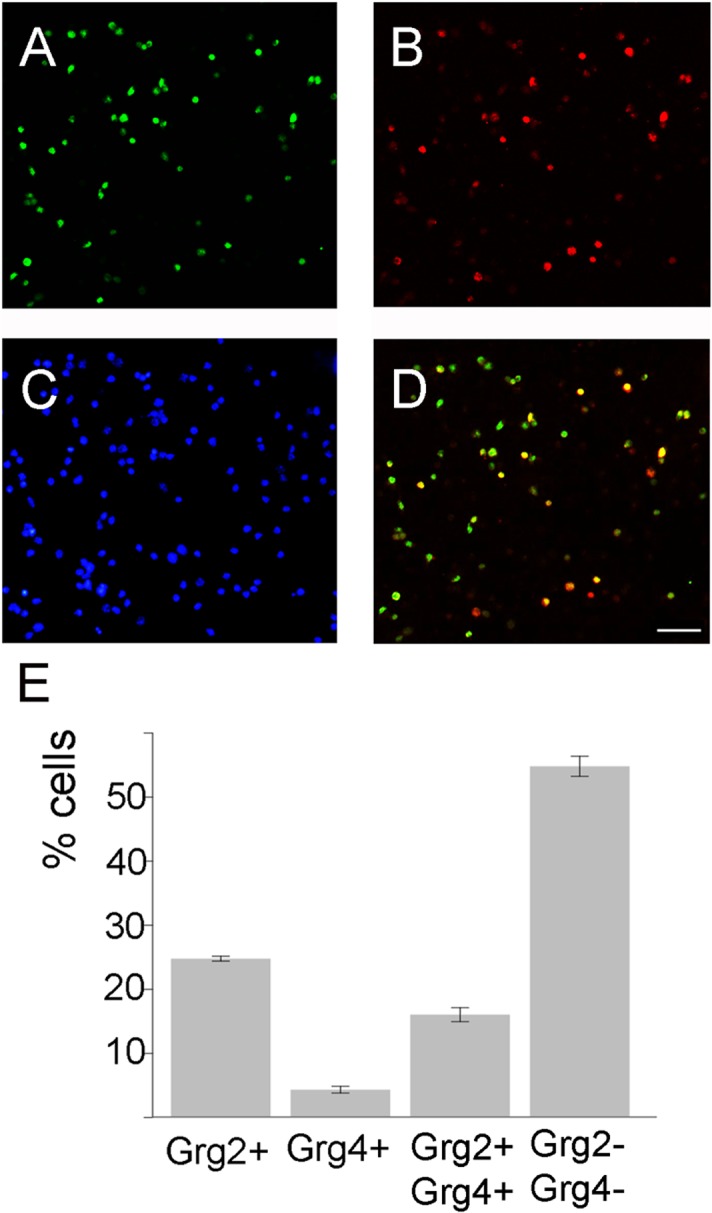
immunofluorescence on primary MGE cells from E13.5 embryonic brain cultured with an anti-Grg2 antibody (A, D green) and an anti-Grg4 antibody (B, D red). The nuclei were detected with DAPI (C,D). (E) Graph showing the percentage of Grg2+, Grg4+, Grg2+/GRG4+ and Grg2-/Grg2- cells of the MGE. Values represent the mean ± SEM of the immunoreactive cell number, expressed as the percentage of total cell counts from 3 independent experiments. Statistical analyses were performed using Student's t-test. *P<0.05. Scale: 50 μm.

These results showed that in several structures different combinations of *Grgs* are expressed, yet from the hybridization experiments it was not possible to conclude if different cells within a structure express different *Grgs* or if *Grgs* are coexpressed in the same cell. This is particularly apparent in the case of *Grg2* and *Grg4* expression domains. To clarify this issue we used double immunofluorescence on sections of E13.5 embryonic brain with antibodies that are specific for Grg2 and Grg4 and do not cross-react with other members of the Grg family. However, when we tried to count different cell populations, as the MGE cells in the SZV and especially in the MZ are very small and packed and Grg4+ cells are few, we could not clearly discriminate between a single Grg4+ cell and a Grg2+/ Grg4+ cell, thus we chose to perform double immunofluorescence on dissociated cells because with this approach Grg4+ cells are easily distinguished from Grg2+/ Grg4+ and hence, our results were more reliable. As it is shown in Fig 10 approximately 40,8% of the cells of the MGE expressed Grg2 and 20,4% Grg4 (Fig 10); these represent cells of the VZ and the SVZ as Grg2 and Grg4 are not expressed in the VZ of the MGE. Notably, within the Grg2 expressing cell population at least two subpopulations can be characterized based on the levels (high or low) of expression (Fig 10A). In addition, a significant percentage of the cells expressed both corepressors (approximately 16,0%, Fig 10), 4,3% of the cells expressed only Grg4 while 20% of the cells expressed only Grg2; interestingly Grg2 expression levels do not correlate with the expression of Grg4, thus in the population expressing both corepressors, cells may express either high or low levels of Grg2 (Fig 10A–10D).

## Discussion

Transcriptional regulation lies at the heart of the developmental processes that operate in the telencephalon during the proliferation and patterning of the neuronal precursors as well as during specification, migration and differentiation of the post-mitotic neurons (for review see [1–3]). Detailed expression studies of genes encoding TFs along with the analysis of mouse embryos bearing mutations, have unravelled a “molecular code” in which distinct progenitor domains or telencephalic structures are characterized based on the combination of the TFs expressed [1–3, 54]. Nevertheless, relatively little is known about the expression and the role of cofactors, proteins that interact with TFs modifying their activity [55,56]. The members of the *Gro/TLE/Grg* gene family encode transcriptional corepressors that have been implicated in several developmental processes and act either directly, by interacting with TFs, or indirectly, by modifying histone acetylation or chromatin structure [20–23, 25–27].

In this work we performed a detailed comparative analysis of the spatiotemporal expression of the *Grg1-4* family members during embryonic neurogenesis in the developing telencephalon as very little is known from previous work [43–47]. *Grg1-4* presented a unique, complex yet overlapping expression pattern; *Grg1* and *Grg3* are mainly detected in the proliferative zones of the telencephalon, *Grg2* mainly in the subpallium and *Grg4* mainly in subpallial post mitotic neurons. *Grg1-4* are located in different chromosomes in the mouse genome and phylogenetic analysis has shown that these genes are the result of three independent duplication events occurred during evolution; this is reflected in the sequences of the proteins they encode, with Grg1 and Grg4 being the more closely related members and Grg2 the most divergent of all four [26, 29]. Interestingly, the expression patterns of *Grg1* and *Grg4* in the developing telencephalon show little if any similarity; only *Grg1* and *Grg3* patterns show extensive overlap. These results suggest that different regulatory elements used by the full length Grg family members have independently of the coding sequences evolved; this may correlate with the development of specific cell types or structures of the brain. *Grg1*, *Grg2* and *Grg4* expression has also been studied in the developing telencephalon of *Xenopus tropicalis* [51] while in *Xenopus laevis* only *Grg4* expression has been analysed [57,58]; in these studies and at equivalent developmental stages, the expression of the *Xenopus tropicalis Grg1* was broadly detected in all telencephalic areas while the expression of *Xenopus tropicalis Grg2* was detected in the ventral telencephalon [51]. However, the expression of these genes was not confined in a specific zone of the telencephalon as we observe in our experiments in the mouse embryo in which *Grg1* expression is restricted in the VZ and *Grg2* in the SVZ and mantle zone [51]. In the developing telencephalon of *Xenopus* and at equivalent stages *Grg4* was broadly expressed, in our experiments however, we do not observe broad distribution of the *Grg4* mRNA analysed [57]. Several studies based on the analysis of the expression of sets of regulatory genes have established that the tetrapod telencephalon is organized into specific, comparable and molecularly distinct divisions and subdivisions [58–60]; however differences, such as the differences we observe in the *Grg* expression patterns, have been described between *Xenopus* and mouse for several genes and are considered to reflect modifications related to the anamniote to amniote transition [60–63].

Gro/TLE/Grg proteins have been mainly regarded as constitutively, uniformly distributed proteins with context-specific repression, regulated to a large extent by the availability of their partners. However, studies performed in the developing pancreas and spinal cord have challenged this view [42, 49]. For instance in the developing spinal cord, all four full-length Grg members are broadly expressed in overlapping domains and their interactions with homeodomain TFs are crucial for patterning [42]. Moreover, during pancreas development *Grg1-4* present overlapping expression patterns in pancreatic progenitors and their expression in the course of endocrine cell maturation is restricted to different cell types [49]. In addition, the association between Foxg1 and Grg2 has been implicated in the proper patterning of the ventral telencephalon in *Xenopus* [51].

Our analysis suggests that, as in the developing pancreas, in the murine telencephalon Grg1-4 may have distinct functions as their expression is spatially regulated and their patterns only partially overlapping. For instance in the VZ of the ventral telencephalon, *Grg3* is uniformly expressed, however, in the VZ of the preoptic area *Grg4* is also expressed, while in the ganglionic eminences various levels of *Grg1* are detected, with the lowest in the VZ of the rostroventral MGE and the highest in the VZ of the LGE; hence differential expression of Grg members within the VZ of the supallium may be part of their positional information; thus the combination Grg3/4 and Grg1/3 may specify PO and ganglionic eminences respectively, while within the ganglionic eminences, the low-to-high ratio of Grg1/Grg3 may specify MGE and LGE respectively. Likewise, the SVZ, in the PO is characterized by the expression of Grg1/2, in the MGE by the expression of Ggr2/4 and in the LGE by the expression of Grg3/4. Within the mantle zone, similar distinct, yet more complicated patterns are observed for Grg2 and Grg4. For example in the preoptic mantle both Grg2 and Grg4 are expressed, within the MGE mantle Grg2 and Grg4 are expressed with specific yet overlapping patterns, while in the LGE mantle only Grg4 is observed. Overall, the comparative analysis of the patterns of Grg1-4 along with *Gsh2*, *Dlx2*, *Nkx2*.*1*, *Lhx6*, *Lhx7/8* and *Arx* revealed that distinct progenitor domains or structures within the telencephalon expressed different combinations of Grg repressors, forming a “thus Grg-mediated repression map”.

What are the implications of this “Grg-mediated repression map” for the patterning and the specification of the telencephalon? Given that several of the key TFs implicated in the development of the telencephalon interact with Grg family members (for example members of the Nkx family and Arx; 49–51) the differential expression of Grgs within the expression domain of a TF could differentially affect its activity; for instance in the MGE Nkx2.1 acts in the VZ as a repressor while in the SVZ along with Lhx6, as an activator [64]; Grg1 and Grg3 are expressed in the VZ of the MGE and Grg2 in the SVZ zone, the differential interaction of these Grgs with Nkx2.1 expressed in these zones, may be part of the molecular mechanism that underlies the regulation of its activity. Our immunofluorescence experiments showed that in the MGE, based on the expression Grg2 and Grg4 at least five distinct subpopulations can be characterized; a weak Grg2+/Grg4+ subpopulation, a strong Grg2+/Grg4+, a weak Grg2+, a strong Grg2+ and a Grg4+ subpopulation. In this territory Arx, that has been shown to interact with Grg2 in the pancreas, is also widely expressed, however in this context the levels of the modulation of Arx may vary depending upon the expression (high or low) of Grg2 and the presence or not of Grg4. Alternatively, different members of the Grg family may interact with different factors differentially affecting either histone acetylation or chromatin structure [21, 23, 25–27, 32, 33].

In summary we propose that within a progenitor domain or structure, distinct Grg combinations are expressed and differentially modify the action of transcription factors spatially restricted in this domain or structure, therefore influencing its patterning or specification; however to understand the impact of Grg-mediated repression during embryonic neurogenesis of the telencephalon the underlying molecular mechanisms that operate need to be elucidated.

## Supporting information

S1 Fig In situ hybridization on sagittal sections through the embryonic telencephalon of E13.5 mouse embryos hybridized with a sense RNA probe of Grg1 (A), Grg3 (B), Grg2 (C) and Grg4 (D) m: MGE, l: LGE, l: LGE, s: septum. Scale: 500 μm.
(TIF)
