# Supplementary figures and images for: Spatial distribution of the full-length members of the Grg family during embryonic neurogenesis reveals a “Grg-mediated repression map” in the mouse telencephalon
Source: PLoS One. 2018 Dec 20;13(12):e0209369. doi: 10.1371/journal.pone.0209369 (PMC6301688; doi:10.1371/journal.pone.0209369)

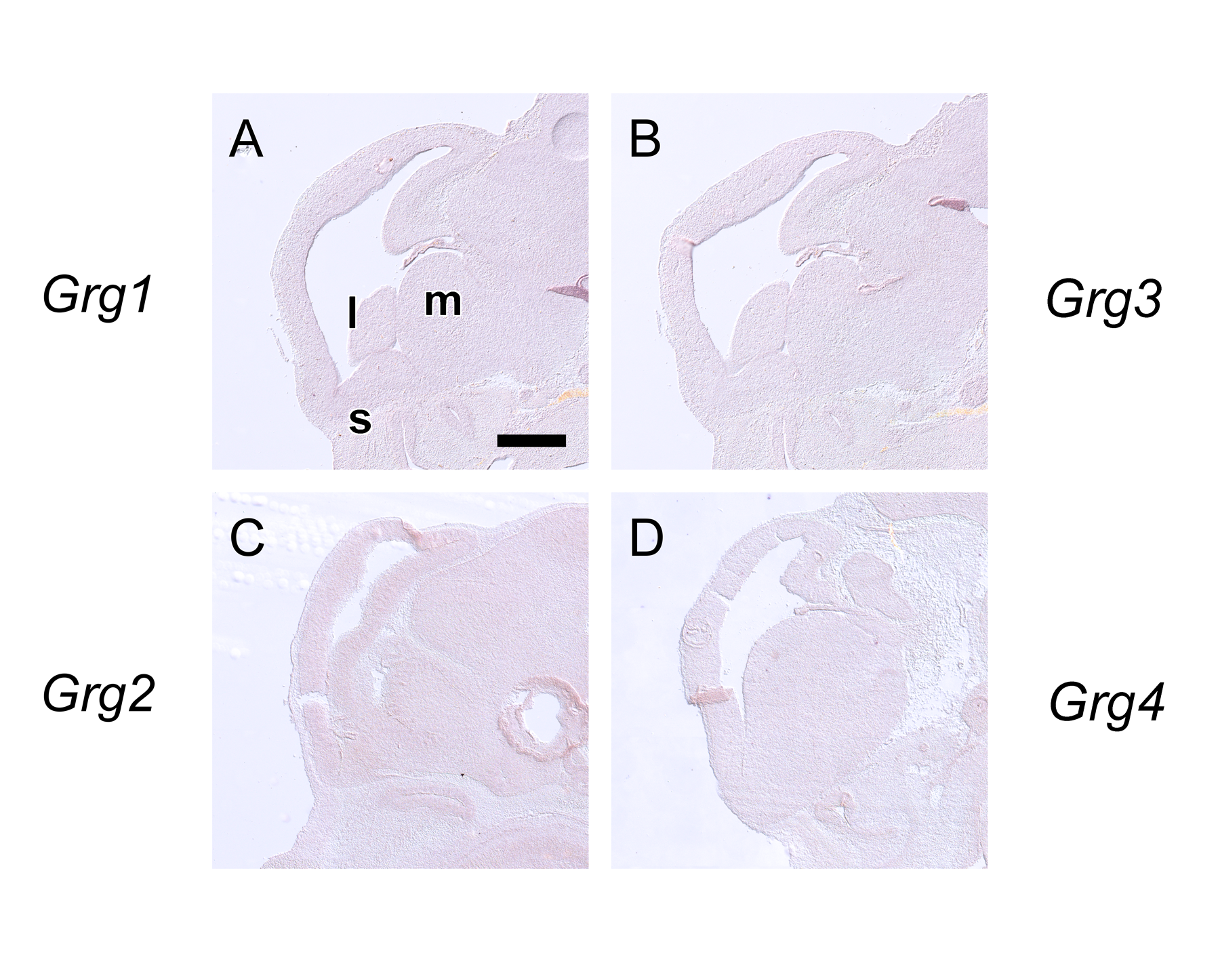

Supplement: S1 Fig — In situ hybridization on sagittal sections through the embryonic telencephalon of E13.5 mouse embryos hybridized with a sense RNA probe of Grg1 (A), Grg3 (B), Grg2 (C) and Grg4 (D) m: MGE, l: LGE, l: LGE, s: septum. Scale: 500 μm. (TIF) [file pone.0209369.s001.tif]
